# Differential metabolites in cirrhotic patients with hepatitis B and muscle mass loss

**DOI:** 10.3389/fnut.2023.1068779

**Published:** 2023-02-16

**Authors:** Xuechun Liu, Lei Han, Shenghua Bi, Xueli Ding, Qi Sheng, Yueping Jiang, Ge Guan, Qinghui Niu, Xue Jing

**Affiliations:** ^1^Department of Gastroenterology, The Affiliated Hospital of Qingdao University, Qingdao, Shandong, China; ^2^Department of Nutrition, The Affiliated Hospital of Qingdao University, Qingdao, Shandong, China; ^3^Liver Disease Center, The Affiliated Hospital of Qingdao University, Qingdao, Shandong, China

**Keywords:** hepatitis B virus-related liver cirrhosis, muscle mass loss, sarcopenia, differential metabolites, amino acid metabolism, gut-liver-muscle axis

## Abstract

**Background:**

Sarcopenia leads to complications (infections, hepatic encephalopathy and ascites) and poor overall survival in patients with cirrhosis, in which the phenotypic presentation is loss of muscle mass. This study aimed to reveal the metabolic profile and identify potential biomarkers in cirrhotic patients with hepatitis B virus and muscle mass loss.

**Method:**

Twenty decompensated cirrhotic patients with HBV and muscle mass loss were designated Group S; 20 decompensated cirrhotic patients with HBV and normal muscle mass were designated Group NS; and 20 healthy people were designated Group H. Muscle mass loss was defined as the skeletal muscle mass index less than 46.96 cm^2^/m^2^ for males and less than 32.46 cm^2^/m^2^ for females. Gas chromatography–mass spectrometry was used to explore the distinct metabolites and pathways in the three groups.

**Results:**

Thirty-seven metabolic products and 25 associated metabolic pathways were significantly different in the Group S patients from Group NS patients. Strong predictive value of 11 metabolites (inosine-5′-monophosphate, phosphoglycolic acid, D-fructose-6-phosphate, N-acetylglutamate, pyrophosphate, trehalose-6-phosphate, fumaric acid, citrulline, creatinine, (r)-3-hydroxybutyric acid, and 2-ketobutyric acid) were selected as potential biomarkers in Group S patients compared with Group NS patients. Two pathways may be associated with loss of muscle mass in patients with liver cirrhosis: amino acid metabolism and central carbon metabolism in cancer.

**Conclusion:**

Seventy differential metabolites were identified in patients who have liver cirrhosis and loss of muscle mass compared with patients who have cirrhosis and normal muscle mass. Certain biomarkers might distinguish between muscle mass loss and normal muscle mass in HBV-related cirrhosis patients.

## Introduction

Sarcopenia is a progressive and diffuse loss of muscle mass, strength, and function. Sarcopenia can be classified into age-related sarcopenia and secondary sarcopenia, the latter being caused by chronic diseases (1). The prevalence of sarcopenia in patients liver cirrhosis is about 40–70% (2). A consensus definition for sarcopenia in these patients is loss of muscle mass (3, 4), which is associated with poor prognosis, including reduced quality of life, increased risk of infection, prolonged hospitalization, and increased rate of mortality (5, 6). Previous studies have focused on the pathology of sarcopenia and its associated factors. In patients with cirrhosis, abnormalities of the gut-liver-axis may be associated with the development of sarcopenia (4, 7). Cirrhosis itself leads to skeletal muscle disorders through several pathways: altered catabolic state, altered protein metabolism, and impaired hepatic ammonia clearance (8), and the metabolic disorders may be the root cause of the low muscle mass. Metabolomics, an emerging discipline, is frequently used to investigate pathophysiological processes involved in disease progression and the identification of new diagnostic or prognostic biomarkers (9, 10). Patients with alcoholic liver disease and cirrhosis have alterations in metabolites, as in nucleic acids and amino acids (11). Patients with non-alcoholic steatohepatitis (NASH)-related cirrhosis have an increased risk of sarcopenia because of the additive effects of insulin resistance and chronic systemic inflammation (12). Cholestasis-predominant liver diseases, such as primary sclerosing cholangitis, have elevated serum bile acid levels, which may induce skeletal muscle atrophy through the bile acid receptor, TGR5, that is expressed in healthy muscles (13, 14). However, the differential metabolites in hepatitis B virus (HBV)-related decompensated liver cirrhosis with loss of muscle mass vs. normal muscle mass are mostly unknown. Therefore, our study aimed to characterize the metabolic profile and identify potential biomarkers of muscle mass loss in HBV-related decompensated liver cirrhosis.

## Materials and methods

### Study population

Patients with HBV-related liver cirrhosis were recruited between August 2021 and June 2022 at the Affiliated Hospital of Qingdao University. All participants provided written informed consent. The diagnosis of cirrhosis was made on the combination of clinical and laboratory features or by liver histopathology (15, 16). Decompensated cirrhosis was defined as patients symptomatic with ascites or gastrointestinal bleeding, or so on (17, 18). Patients were excluded for (1) causes of cirrhosis other than chronic HBV infection, such as alcoholic cirrhosis, autoimmune cirrhosis, and others, (2) age over 65 years, (3) acute-chronic and acute liver failure, (4) other diseases that can lead to secondary muscle depletion, such as chronic diseases of the heart, lungs, kidneys, or brain, and malignant tumors (5) neurodegenerative or muscle degenerative diseases, (6) perioperative patients, (7) patients without computed tomography (CT) scan within 3 months, (8) the Nutrition Risk Screening 2002 score of the patients was over 3 points (19), and (9) patients who have exercise habits with Physical Activity Rating Scale-3 > 19 points (20). Patients excluded from the healthy control group were those with metabolic diseases (diabetes, thyroid disorder, and others) and other chronic diseases, according to their ultrasound and laboratory assessments.

### Skeletal muscle measurement and diagnostic criteria

Skeletal muscle mass index (SMI) is defined as the ratio of lean tissue area to body height. Lean tissue area is the skeletal muscle area (SMA) calculated according to CT readings at the level of the third lumbar vertebra (L3). Muscle attenuation (MA), which is associated with muscle density and intramuscular lipid content, is the mean Hounsfield unit (HU) of the entire SMA (21). All the recruited patients had abdominal CT scans at admission. Two sequential transverse CT images at the level of L3 were analyzed with SliceOmatic V5.0 software (Tomovision, Montreal, QB, Canada), which enables specific tissue demarcation using HU thresholds. The CT HU thresholds were −29 to 150 for quantifying muscle mass; −150 to −50 for visceral adipose tissue; and −190 to −30 for subcutaneous fat tissue. The formula for SMI was SMI (cm^2^/m^2^) = SMA (cm^2^)/height (m^2^). Muscle mass loss was defined as SMI less than 46.96 cm^2^/m^2^ for males and less than 32.46 cm^2^/m^2^ for females (22).

### Clinical and laboratory assessments

Morning fasting blood was collected and centrifuged at 3000×g for 15 min. The plasma was stored at −80°C until the gas chromatography–mass spectrometry (GC–MS) analysis was conducted.

### Preparation of samples

Samples were thawed at room temperature before analysis. One-hundred-and-fifty microliters of samples were placed in a 1.5-mL Eppendorf tube with L-2-chlorophenylalanine (0.06 mg/ml) dissolved in methanol as internal standard, and the tube was vortexed for 10 s. An ice-cold mixture of methanol and acetonitrile (vol:vol, 2:1) was added, and the mixtures were vortexed for 30 s. The whole samples were extracted by ultrasound for 10 min in ice-water bath and placed at −20°C for 30 min. The extract was centrifuged at 4°C (13,000 rpm) for 10 min. 150 μl of supernatant in a glass vial were dried in a freeze-concentration centrifugal dryer, and 80 μl of 15 mg/ml methoxyamine hydrochloride in pyridine was added. The mixture was vortexed vigorously for 2 min and incubated at 37°C for 90 min. 50 μl of bistrifluoroacetamide with 1% trimethylsilyl chloride and 20 μl n-hexane were added into the mixture, which was vortexed vigorously for 2 min and then derivatized at 70°C for 60 min. The samples were placed at ambient temperature for 30 min before GC–MS analysis.

### Gas chromatography–mass spectrometry analysis

The derivatized samples were analyzed on an Agilent 7890B gas chromatography system coupled to an Agilent 5977A MSD system (Agilent Technologies Inc., Santa Clara, CA, United States). A DB-5MS fused-silica capillary column (30 m × 0.25 mm × 0.25 μm) (Agilent J & W Scientific, Folsom, CA, USA) was used to separate the derivatives. Helium (>99.999%) was used as the carrier gas at a constant flow rate of 1 ml/min through the column. The injector temperature was maintained at 260°C. The initial oven temperature was 60°C, held at 60°C for 0.5 min, ramped to 125°C at a rate of 8°C/min, to 210°C at a rate of 5°C/min, to 270°C at a rate of 10°C/min, to 305°C at a rate of 20°C/min, and finally held at 305°C for 5 min. The temperature of MS quadrupole and ion source (electron impact) were set to 150°C and 230°C, respectively. The collision energy was 70 eV. Mass spectrometric data were acquired in a full-scan mode (m/z 50–500), and the solvent delay time was set to 5 min. The quality control samples (QC) were injected at regular intervals (every 10 samples) throughout the analytical run to provide a set of data from which repeatability could be assessed.

### Statistical analysis

SPSS 26.0 software (International Business Machines Corp., Chicago, IL, United States) was used for the statistical analyses. Mean ± standard deviation was used in quantitative variables, and the significance was determined with a Student’s t-test. Non-normally distributed variables are expressed as a median and interquartile range, and the significance was determined using a Mann–Whitney U test. *p* value <0.05 indicated statistically significant differences. A systematic analysis of the metabolites was identified and confirmed *via* searching the Kyoto Encyclopedia of Genes and Genomes (KEGG) database.^1^

### Ethics statement

The study protocol was approved by the ethics board of the affiliated hospital of Qingdao University (No. QYFYWZLL26461). We also registered this study on ClinicalTrials.gov (NCT05041348).

## Results

### Baseline clinical characteristics of patients

The baseline clinical characteristics of the patients with HBV-related liver cirrhosis are summarized in Table 1. Twenty decompensated cirrhotic patients with HBV and muscle mass loss (Group S), 20 decompensated cirrhotic patients with HBV and normal muscle mass (Group NS), and 20 healthy people (Group H) were included in this study. There were no significant differences in BMI and the model for end-stage liver disease score (MELD score) among the groups (*p* > 0.05). The SMI and SMA in Group S were significantly lower than in Group NS (*p* < 0.05). Alanine transaminase and alkaline phosphatase values were significantly higher in Group NS than in Group S (*p* < 0.05).

**Table 1:** Demographic and clinical characteristics of the study participants.

	Cirrhosis with muscle mass loss (Group S) *N* = 20	Cirrhosis with normal muscle mass (Group NS) *N* = 20	Healthy (Group H) *N* = 20	Value of *p*
Age	48 (29–62)	49.8 (32–61)	31 (22–42)	**<0.05**
Gender (*n*) Male/Female	16/4	14/6	9/11	
BMI	23.17 (21.84–25.92)	25.63 (22.54–28.10)	23.45 (20.85–24.75)	0.06
Child-Pugh stage (*n*)				
A	8	6		
B	9	6		
C	5	8		
MELD score	12.10 ± 3.42	12.55 ± 4.07		0.07
SMI	40.29 ± 4.85	50.32 ± 6.60		**<0.05**
SMA	121.06 ± 18.02	144.71 ± 27.45		**<0.05**
MA	39.81 ± 5.45	43.17 ± 5.20		0.053
SFA	99.29 ± 55.24	133.36 ± 64.75		0.081
VFA	83.13 ± 55.53	78.21 ± 55.17		0.780
Pre-albumin	75.59 ± 34.35	96.7 ± 58.69		0.174
Total protein	59.43 ± 8.83	60.97 ± 10.40		0.618
Albumin	31.60 ± 5.94	31.14 ± 6.63		0.817
Albumin/ Globulin	1.13 ± 0.26	1.07 ± 0.21		0.448
ALT	19 (14–29.75)	29.5 (24.5–55)		**<0.05**
AST	27 (19.75–41.75)	34 (28–60.5)		0.053
GGT	16.5 (12.25–40.75)	30.5 (19.75–68.03)		0.064
ALP	76.5 (51–89)	101 (70–142)		**<0.05**
Total bilirubin	29.47 ± 17.08	40.76 ± 27.83		0.132
Direct bilirubin	9.05 (6.68–11.13)	10.55 (5.53–27.95)		0.358
Creatinine	78.45 (72.5–89)	74.85 (68.25–95.25)		0.482
BUN	5.17 (3.90–7.52)	4.58 (3.67–6.10)		0.552
D-dimer	855 (322.5–1765)	525 (247.5–962.5)		0.133
PT	16.66 ± 2.52	15.76 ± 2.76		0.291
INR	1.41 ± 0.23	1.35 ± 0.24		0.421
Fibrinogen	2.00 ± 0.52	1.85 ± 0.52		0.432

BMI, body mass index; SMI, skeletal muscle mass index; SMA, skeletal muscle area; MA, muscle attenuation; VFA, visceral adipose tissue; SFA, subcutaneous fat tissue; ALT, alanine transaminase; AST, aspartate aminotransferases; GGT, gamma glutamyl transferase; ALP, alkaline phosphatase; BUN, blood urea nitrogen; PT, prothrombin time; INR, International Normalized Ratio. The bold values means: *p* value <0.05 indicated statistically significant differences.

### Overall metabolomics analysis of samples

**Figure 1:**
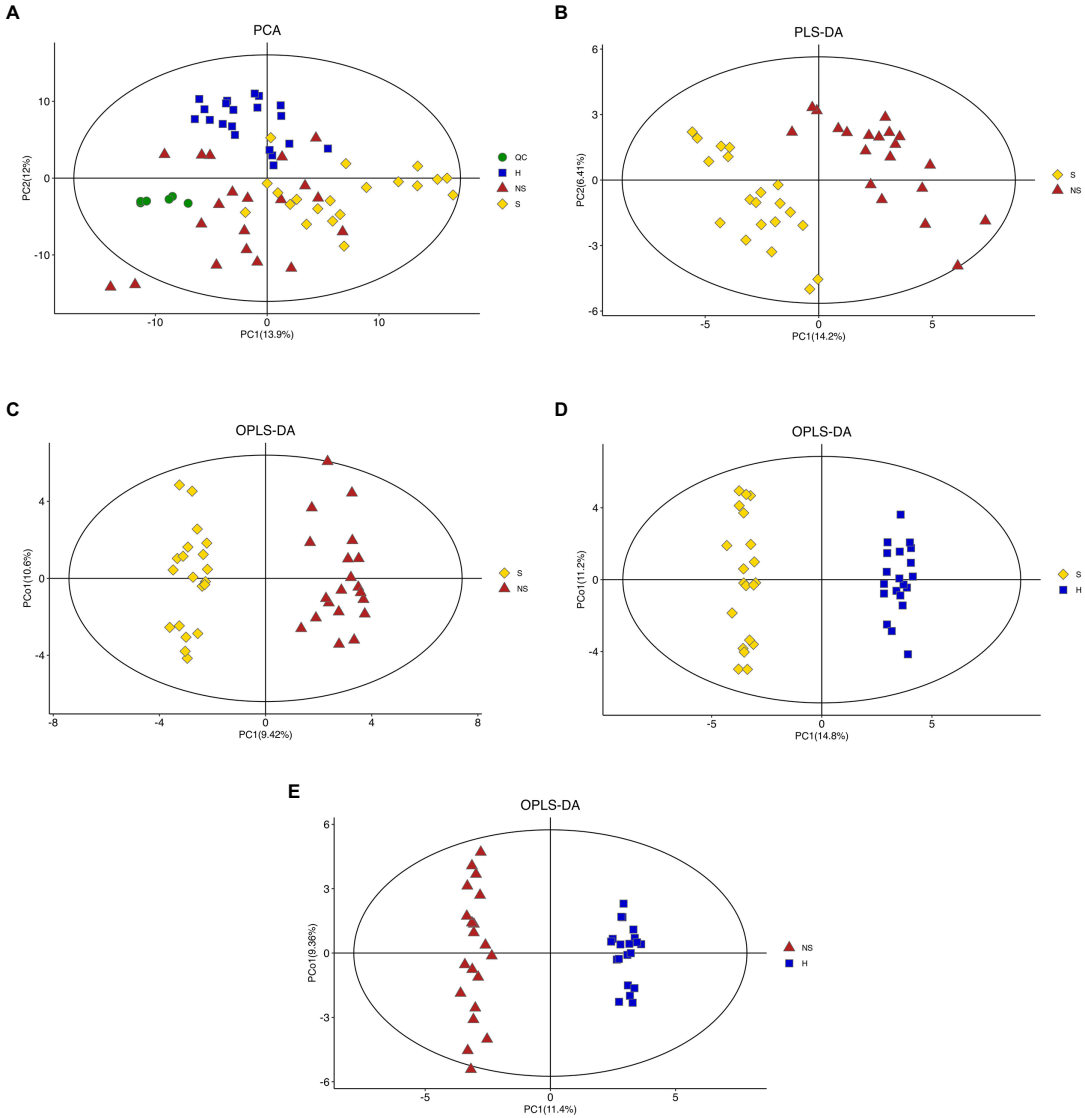
Score plot. **(A)** Unsupervised principal component analysis (PCA) plots of all groups (QC, Quality control samples; H, Health control group; S, HBV-related decompensated liver cirrhosis patients with muscle mass loss; NS, liver cirrhosis patients with normal muscle mass), **(B,C)** Partial least-squares-discriminant analysis (PLS-DA) and orthogonal partial least-squares discriminant analysis (OPLS-DA) score plots of Group S and Group NS, **(D)** OPLS-DA score plots of Group S and Group H, and **(E)** OPLS-DA score plots of Group NS and Group H.

The unsupervised principal component analysis (PCA) revealed that the healthy control group (H) could be distinguished from other groups (Figure 1A). The QC samples clustered tightly, indicating stability of the method. PCA was used to reveal the overall distribution among the samples and the stability of the whole analysis. To exclude possible confounding variables, partial least-squares-discriminant analysis (PLS-DA) and orthogonal partial least-squares discriminant analysis (OPLS-DA) were used to distinguish the metabolites that differed between groups (Figures 1B,C). Although the distribution of Group S samples overlapped with the distribution of Group NS samples, the PLS-DA and OPLS-DA (R2Y = 0.958, Q2 = 0.683, and R2Y = 0.958, Q2 = 0.715, respectively) indicated high cross-validation predictability and goodness-of-fit. The OPLS-DA also revealed significant discrimination between Group S and Group H (Figure 1D, R2Y = 0.988 and Q2 = 0.897) and between Group NS and Group H (Figure 1E, R2Y = 0.99 and Q2 = 0.834.). The combined results indicated reliable differences among the three groups.

### Metabolites profiling in the three groups

**Figure 2:**
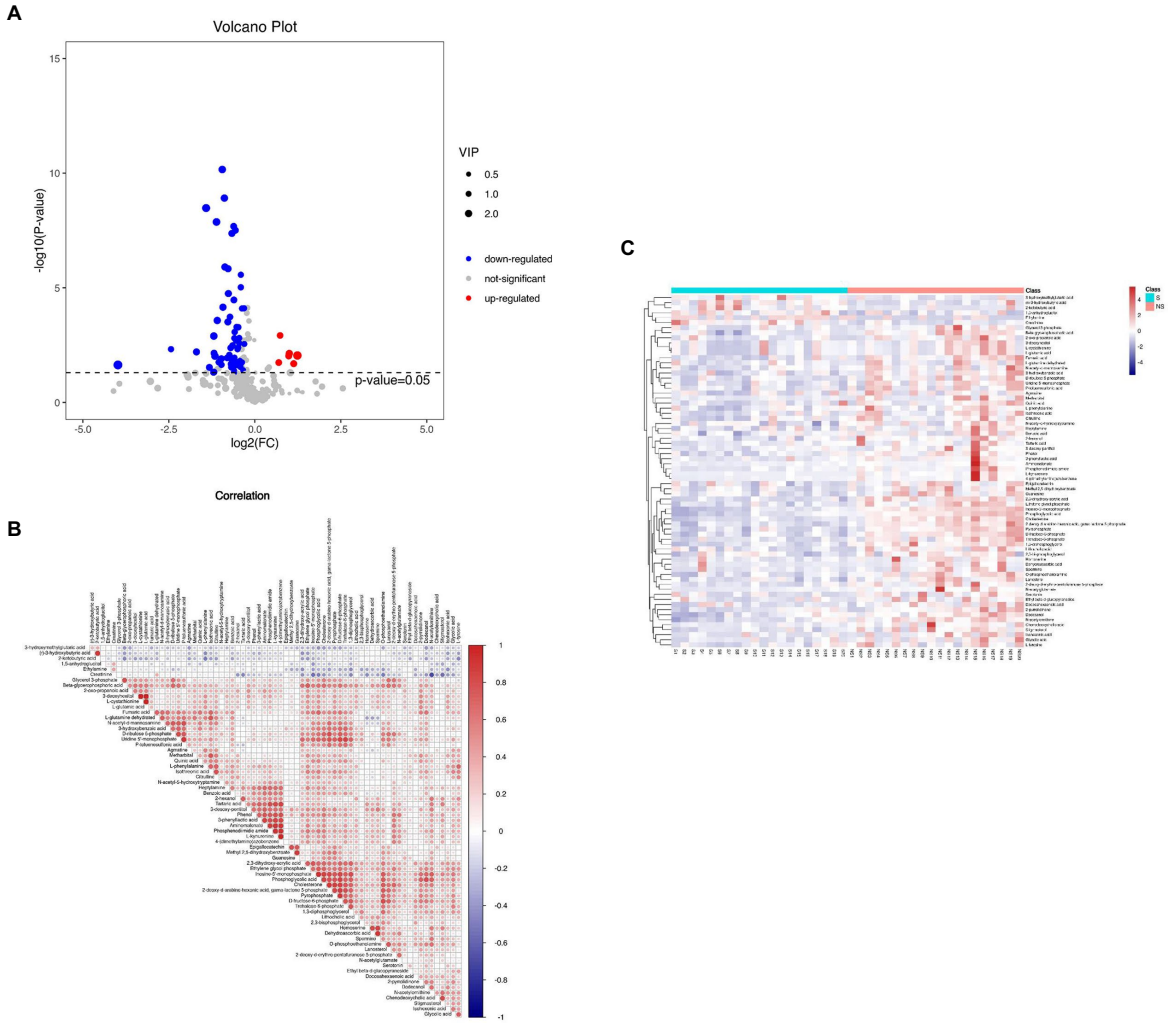
**(A)** The volcano plot of Groups S and NS, **(B)** Correlation analysis of metabolites in Group S and NS, and **(C)** The heatmap of the correlation analysis revealed the expression level of metabolites in Groups S and NS.

A total of 162 volatile metabolites were identified in the three groups, with variable importance on projection (VIP) scores >1 and *p* values <0.05. With Group S and Group H combined, 102 differential metabolites were detected. Ninety-three metabolites were found in Group NS and Group H. Information of the metabolites is illustrated in Supplemental Figure 1. To identify potential biomarkers that may be related to muscle mass loss, we compared the metabolite profile between Group S and Group NS. Seventy differential metabolites (consisting mainly of organic acids and derivatives, lipids, and lipid-like molecules, and organic oxygen compounds) were detected with VIP >1 and *p* < 0.05. The visualized volcano plot (Figure 2A) showed the correlation of the metabolites between Group S and Group NS, including elevated levels of ethylamine, (r)-3-hydroxybutyric acid, 3-hydroxymethylglutaric acid, 2-ketobutyric acid, 1,5-anhydroglucitol, and creatinine. The levels of 64 metabolites were downregulated in Group S. Correlation analysis illustrated the relationships among the metabolites (Figure 2B). A heatmap of the correlation analysis revealed the expression levels of the metabolites (Figure 2C).

### Pathways related to liver cirrhosis with muscle mass loss

**Figure 3:**
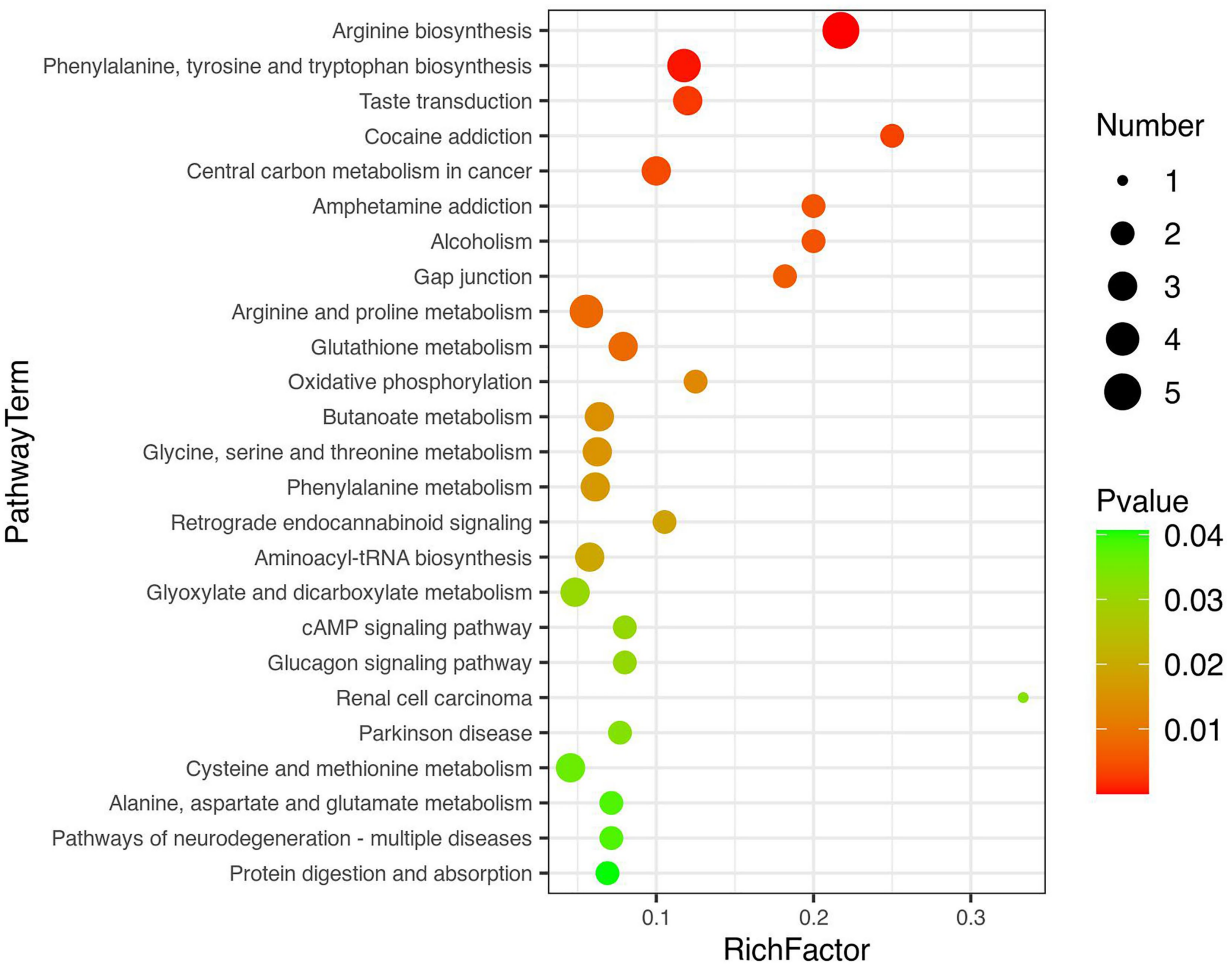
Pathway enrichment and topology analysis.

Twenty-five pathways were defined as disturbed in the plasma of liver cirrhosis patients who had muscle mass loss (10 pathways with *p* values <0.01 and 15 pathways with *p* value <0.05). Thirty-seven differential metabolites were enriched in the disturbed pathways; the corresponding KEGG map of these potential biomarkers, the value of fold change, and other information are presented in Supplemental Table 1. Most of the disturbed pathways were related to amino acid metabolism (Figure 3). Node color is based on the p value (red indicates a higher level of significance), and node radius is determined by the number of differential metabolites in this pathway. The relationships between metabolites and pathways are illustrated in Supplemental Table 2.

### Differential metabolites detected in group S and NS

Among the differential metabolites in the groups, three differential metabolites with enrichment pathways appeared in Group S compared with Group NS. Citrulline (VIP = 1.59, *p* = 0.003) and agmatine (VIP = 1.02, *p* = 0.039) were downregulated in Group S, and 2-ketobutyric acid (VIP = 1.73, *p* = 0.020) was upregulated in Group S. Creatinine (VIP = 1.473, *p* = 0.001) and (r)-3-hydroxybutyric acid (VIP = 2.02, *p* = 0.007) levels were elevated in Group S but not as unique metabolites. Inosine-5′-monophosphate (VIP = 2.24, *p* < 0.001), with the highest VIP score, was decreased in Group S. L-glutamic acid (VIP = 1.45, *p* = 0.028) and L-tyrosine (VIP = 1.16, *p* = 0.011), which appeared in almost all the enrichment pathways, may be the hub of various metabolic pathways related with muscle mass loss. All the results suggested that amino acid metabolism and central carbon metabolism in cancer are associated with muscle mass loss in patients with liver cirrhosis.

### Potential biomarkers selected by receiver operator characteristic curve

**Figure 4:**
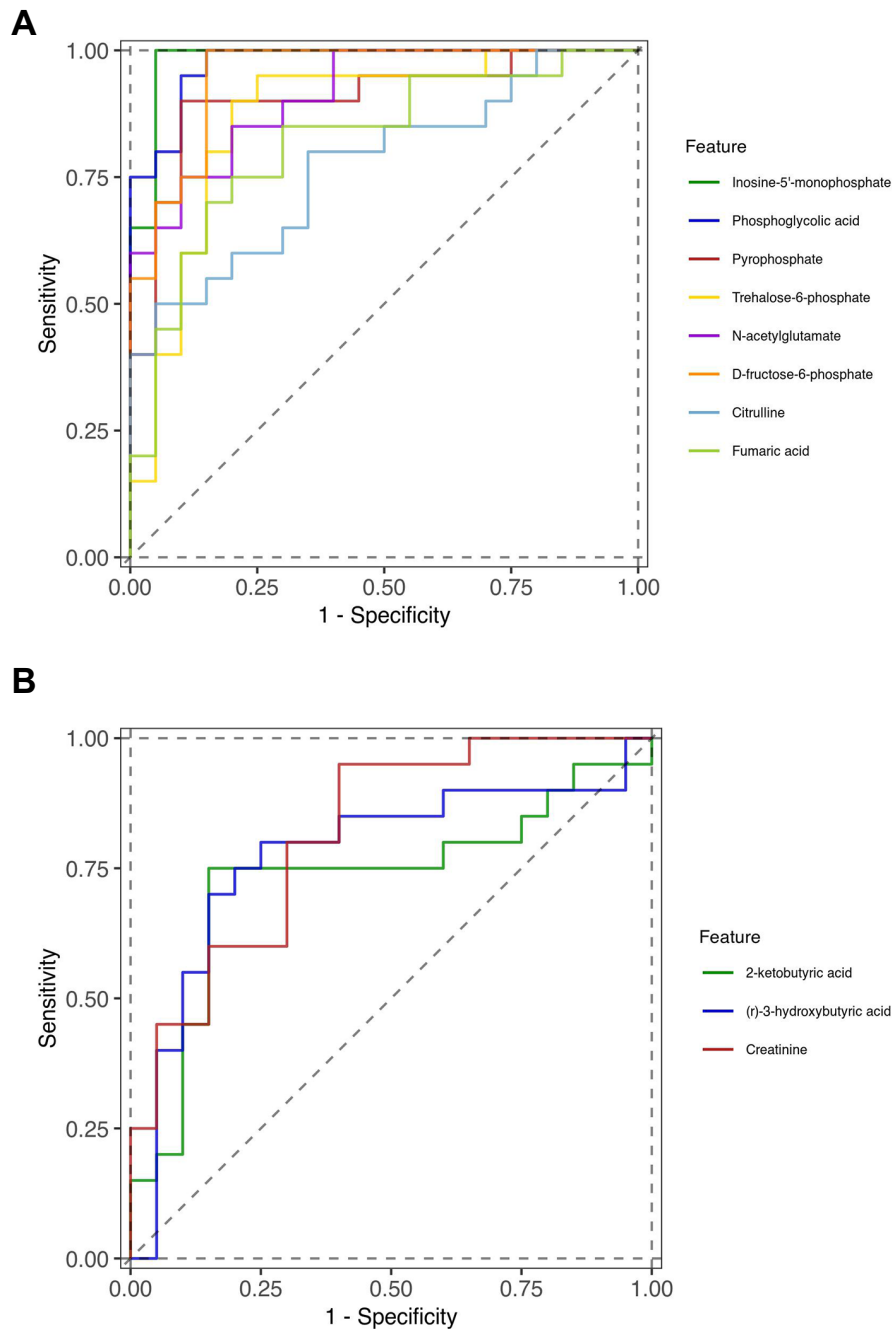
ROC curves of the diagnostic metabolites. **(A)** downregulated metabolites in Group S and **(B)** upregulated metabolites in Group S.

To further identify the key metabolites that could distinguish liver cirrhosis with muscle mass loss from those with normal muscle mass, ROC curves were analyzed (Figures 4A,B). The area under the curve (AUC) was used to assess the potential diagnostic value: inosine-5′-monophosphate, 0.9825 (95% confidence interval (CI) 0.946–1.000), phosphoglycolic acid, 0.975 (95% CI 0.937–1.000), D-fructose-6-phosphate, 0.95 (95% CI 0.887–1.000); N-acetylglutamate, 0.9125 (95% CI 0.829–0.996); pyrophosphate, 0.905 (95% CI 0.806–0.988); trehalose-6-phosphate, 0.87 (95% CI 0.752–1.000); fumaric acid, 0.82 (95% CI 0.687–0.953); citrulline, 0.7725 (95% CI 0.627–0.918); creatinine, 0.815 (95% CI 0.684–0.946); (r)-3-hydroxybutyric acid, 0.775 (95% CI 0.618–0.932); 2-ketobutyric acid, 0.7275 (95% CI 0.559–0.896). The AUCs were >0.7, indicating the diagnostic accuracy of these metabolites.

## Discussion

In this study, we identified by GC–MS analysis the differential metabolites in cirrhotic patients with HBV and muscle mass loss. The results revealed clear separation of three groups (decompensated liver cirrhosis with muscle mass loss; decompensated liver cirrhosis with normal muscle mass; and healthy people). To the best of our knowledge, this is the first study that analyzed the plasma metabolic characteristics of patients with HBV-related liver cirrhosis and associated loss of muscle mass. We found that there were two pathways that may be associated with loss of muscle mass in patients with liver cirrhosis: amino acid metabolism, and central carbon metabolism in cancer. Moreover, inosine-5′-monophosphate, phosphoglycolic acid, D-fructose-6-phosphate, N-acetylglutamate, pyrophosphate, trehalose-6-phosphate, fumaric acid, citrulline, creatinine, (r)-3-hydroxybutyric acid, and 2-ketobutyric acid were found potential biomarkers in HBV-related liver cirrhosis with muscle mass loss.

Arginine is a semi-essential amino acid that participates in the urea cycle, the nitric oxide (NO) cycle, and muscle protein synthesis. Citrulline, as a non-essential amino acid, can be metabolized to arginine in the urea and NO cycles and can control the delivery of arginine to the liver. However, higher levels of circulating arginine induce arginases, which result in rapid arginine clearance. Therefore, citrulline is arginine’s precursor (23, 24). Increased levels of citrullines may be crucial in promoting muscle function (25). Bailey et al. (26) found that citrulline, an effective dietary supplement, is better than arginine in improving oxidative metabolism and exercise performance. Caballero-Garcia et al. (27) reported that muscle strength and endurance tended to increase with citrulline supplementation. In this study, citrulline, as a differential metabolite, was specifically downregulated in liver cirrhosis patients who had muscle mass loss. The corresponding arginine biosynthesis pathway was also found an enrichment pathway with a lower *p* value (*p* < 0.001). These results are consistent with previous research and confirmed that decreased plasma concentrations of citrulline are related to muscle mass loss. More studies to investigate the relationship between citrulline and muscle mass loss in patients with liver cirrhosis are needed.

(R)-3-hydroxybutyric acid, one of the ketone bodies in humans, is produced by the liver during carbohydrate deprivation to provide energy to brain, heart, and muscle cells (26). In the fasting state or with decreased oral intake, ketone bodies are increased to provide energy and regulate lipolysis, proteolysis, and ketogenesis (27–29). Studies of ketone body supplementation in athletes and healthy people found that the supplementation increases metabolic flexibility during exercise and enhances endurance exercise performance (30–32). However, we found that (r)-3-hydroxybutyric acid (VIP >2, and FC >2) were increased in liver cirrhosis patients with muscle mass loss. ROC analysis had an AUC of 0.775, implying high specificity and sensitivity. Liver cirrhosis disturbs energy synthesis and metabolism, which in turn influences the nutritional status (33). In disease states, the utilization efficiency of ketone bodies may decrease. Sasaki et al. (32) found that high concentrations of venous ketone bodies in hepatocellular carcinoma patients were related with low skeletal muscle quality. The relationship between ketone bodies, liver cirrhosis, and muscle mass loss is still ambiguous. Our results provide directions for future research.

Creatinine (VIP =1.47, *p* < 0.05, and AUC = 0.815), detected as a biomarker, was increased in Group S. It is a breakdown product of creatine phosphate in muscle. Hence, under the state of stable renal function and normal nutrition intake condition, creatinine concentration can reflect muscle mass (34, 35). Some researchers combined creatinine and cystatin C as a novel index to evaluate muscle mass and a low serum creatinine level, most likely due to sarcopenia, is associated with a higher mortality rate (36, 37). In our study, we excluded patients with kidney disease that can lead to secondary muscle depletion. The method in our hospital to detect creatinine concentration is the Picric acid method which is different from GC–MS. The concentration of creatinine (μmol/L) in the cirrhotic group with and without loss of muscle mass was 78.45 (72.5–89) and 74.85 (68.25–95.25) respectively. It was consistent with the results of GC–MS. According to our results, creatinine may as a sensitive indicator to predict muscle mass loss.

Inosine-5′-monophosphate (IMP), which participates in purine metabolism, is generated by a *de novo* biosynthetic pathway in the liver (38). IMP can be converted to adenosine diphosphate or guanosine-5′-diphosphate and then to adenosine triphosphate or guanosine trisphosphate (39). IMP is an important indicator of meat quality and freshness in animals (40, 41). However, the role of IMP in humans is rarely mentioned. One study concluded that IMP is in low amount in human skeletal muscle at rest and after low-intensity exercise, but it is formed after moderate and high-intensity exercise (42). We found that IMP was downregulated in patients with muscle mass loss. In the future, IMP, which can increase energy availability and protein synthesis, may be considered for use as a dietary supplement.

In this study, phosphoglycolic acid (VIP >2, *p* < 0.05, and AUC = 0.975), a differential metabolite, was decreased in patients with liver cirrhosis and muscle mass loss. Phosphoglycolic acid can be regulated by phosphoglycolate phosphatase, which is a recently identified mammalian enzyme at the intersection of glucose metabolism, lipogenesis, lipolysis, and cellular nutrient-excess detoxification (43, 44). The relationship between phosphoglycolate phosphatase activity, liver function, and muscle mass was not clear. More studies are needed to determine the function of phosphoglycolic acid in muscle metabolism.

Our study has strengths: First, sarcopenia can be classified into age-related sarcopenia and secondary sarcopenia. Despite advanced cirrhosis, we excluded patients over 65 years old and other diseases that can lead to secondary muscle depletion, such as chronic diseases of the heart, lungs, kidneys, or brain, malignant tumors, and neurodegenerative or muscle degenerative diseases. In our study, patients with a single cause of liver cirrhosis (HBV infection) were studied, which favored increased homogeneity of the study population. Second, our results identified differential metabolites that may lead to liver cirrhosis-related muscle mass loss and, thus, provided directions for future research.

However, the study has limitations: Although, before we started this study, we calculated the skeletal muscle mass index of 101 liver cirrhosis patients at our hospital. 80 patients can be defined as having low muscle mass. The sample size that we calculated according to the prevalence (79.21%) of liver cirrhosis with low muscle mass at our hospital was around 10.22. And we recruited 20 patients from each group. The current sample size is sufficient to illustrate the conclusion of this manuscript. However, this is a single-center study with a small sample size, and the results will need testing on a larger series of patients. Second, we did not screen for branched chain amino acids as differential metabolites; the relationship of branched chain amino acids with sarcopenia seems clear from the results of recent research: some researchers have reported that branched amino acid treatment improves muscle mass of cirrhotic patients with sarcopenia (45). Finally, we only investigated muscle mass loss through imaging features, not muscle strength, and decreased muscle strength is another characteristic of sarcopenia. Thus, the differential expression of metabolites in liver cirrhosis should be a subject of future research.

In conclusion, this is the first analysis of the plasma metabolic characteristics of HBV-related decompensated liver cirrhosis with accompanying loss of muscle mass. Through GC–MS studies, 37 metabolites with 25 pathways were found disturbed in the plasma of patients with cirrhosis. The potential value of 11 metabolites (inosine-5′-monophosphate, phosphoglycolic acid, D-fructose-6-phosphate, N-acetylglutamate, pyrophosphate, trehalose-6-phosphate, fumaric acid, citrulline, creatinine, (r)-3-hydroxybutyric acid, and 2-ketobutyric acid) as biomarkers of muscle mass was identified. Two pathways may be associated with loss of muscle mass in patients with HBV-related decompensated liver cirrhosis, i.e., amino acid metabolism and central carbon metabolism in cancer.

## Data availability statement

The original contributions presented in the study are included in the article/Supplementary material, further inquiries can be directed to the corresponding author.

## Ethics statement

The study protocol was approved by the ethics board of the affiliated hospital of Qingdao University (No. QYFYWZLL26461). We also registered this study on ClinicalTrials.gov (NCT05041348). The patients/participants provided their written informed consent to participate in this study.

## Author contributions

XL and XJ: conceptualization. LH, GG, and QN: methodology. QS: software. SB and XL: formal analysis. XD: investigation. YJ and XJ: data curation and supervision. XL: writing—original draft preparation. XJ: writing—review and editing and project administration. All authors contributed to the article and approved the submitted version.

## Funding

This study was supported by the Chinese Nutrition Society Food and Fermentation Oligopeptide Nutrition Research Fund (no. CNS-FF2019A24).

## Conflict of interest

The authors declare that the research was conducted in the absence of any commercial or financial relationships that could be construed as a potential conflict of interest.

## Publisher’s note

All claims expressed in this article are solely those of the authors and do not necessarily represent those of their affiliated organizations, or those of the publisher, the editors and the reviewers. Any product that may be evaluated in this article, or claim that may be made by its manufacturer, is not guaranteed or endorsed by the publisher.

## Footnotes

^1^ https://www.kegg.jp

## References

[1] Cruz-Jentoft AJ Bahat G Bauer J Boirie Y Bruyère O Cederholm T. Sarcopenia: revised European consensus on definition and diagnosis. Age Ageing. (2019) 48:16–31. doi: 10.1093/ageing/afy169, PMID: 30312372 PMC6322506

[2] Dasarathy S Merli M. Sarcopenia from mechanism to diagnosis and treatment in liver disease. J Hepatol. (2016) 65:1232–44. doi: 10.1016/j.jhep.2016.07.040, PMID: 27515775 PMC5116259

[3] Lai JC Tandon P Bernal W Tapper EB Ekong U Dasarathy S. Malnutrition, frailty, and sarcopenia in patients with cirrhosis: 2021 practice guidance by the American Association for the Study of Liver Diseases. Hepatology. (2021) 74:1611–44. doi: 10.1002/hep.32049, PMID: 34233031 PMC9134787

[4] Tandon P Montano-Loza AJ Lai JC Dasarathy S Merli M. Sarcopenia and frailty in decompensated cirrhosis. J Hepatol. (2021) 75:S147–62. doi: 10.1016/j.jhep.2021.01.025, PMID: 34039486 PMC9125684

[5] Tantai X Liu Y Yeo YH Praktiknjo M Mauro E Hamaguchi Y. Effect of sarcopenia on survival in patients with cirrhosis: a meta-analysis. J Hepatol. (2022) 76:588–99. doi: 10.1016/j.jhep.2021.11.006, PMID: 34785325

[6] Welch N Attaway A Bellar A Alkhafaji H Vural A Dasarathy S. Compound sarcopenia in hospitalized patients with cirrhosis worsens outcomes with increasing age. Nutrients. (2021) 13:659. doi: 10.3390/nu13020659 33670535 PMC7923160

[7] Ponziani FR Picca A Marzetti E Calvani R Conta G del Chierico F. Characterization of the gut-liver-muscle axis in cirrhotic patients with sarcopenia. Liver Int. (2021) 41:1320–34. doi: 10.1111/liv.14876, PMID: 33713524

[8] Traub J Reiss L Aliwa B Stadlbauer V. Malnutrition in patients with liver cirrhosis. Nutrients. (2021) 13:540. doi: 10.3390/nu13020540 33562292 PMC7915767

[9] Bujak R Struck-Lewicka W Markuszewski MJ Kaliszan R. Metabolomics for laboratory diagnostics. J Pharm Biomed Anal. (2015) 113:108–20. doi: 10.1016/j.jpba.2014.12.017 25577715

[10] Schrimpe-Rutledge AC Codreanu SG Sherrod SD McLean JA. Untargeted metabolomics strategies-challenges and emerging directions. J Am Soc Mass Spectrom. (2016) 27:1897–905. doi: 10.1007/s13361-016-1469-y, PMID: 27624161 PMC5110944

[11] Meyer JJ Dreyhaupt J Schwerdel D Ettrich TJ Backhus J Dollinger MM. Blood-based targeted metabolomics discriminate patients with alcoholic liver cirrhosis from those with non-cirrhotic liver damage: an explorative study. Dig Dis. (2022) 40:223–31. doi: 10.1159/000516488, PMID: 33866312

[12] Dong S Zhan ZY Cao HY Wu C Bian YQ Li JY. Urinary metabolomics analysis identifies key biomarkers of different stages of nonalcoholic fatty liver disease. World J Gastroenterol. (2017) 23:2771–84. doi: 10.3748/wjg.v23.i15.2771, PMID: 28487615 PMC5403757

[13] Abrigo J Gonzalez F Aguirre F Tacchi F Gonzalez A Meza MP. Cholic acid and deoxycholic acid induce skeletal muscle atrophy through a mechanism dependent on TGR5 receptor. J Cell Physiol. (2021) 236:260–72. doi: 10.1002/jcp.29839, PMID: 32506638

[14] Sasaki T Kuboyama A Mita M Murata S Shimizu M Inoue J. The exercise-inducible bile acid receptor Tgr5 improves skeletal muscle function in mice. J Biol Chem. (2018) 293:10322–32. doi: 10.1074/jbc.RA118.002733, PMID: 29773650 PMC6028981

[15] Fukui H Saito H Ueno Y Uto H Obara K Sakaida I. Evidence-based clinical practice guidelines for liver cirrhosis 2015. J Gastroenterol. (2016) 51:629–50. doi: 10.1007/s00535-016-1216-y, PMID: 27246107

[16] Yoshiji H Nagoshi S Akahane T Asaoka Y Ueno Y Ogawa K. Evidence-based clinical practice guidelines for liver cirrhosis 2020. J Gastroenterol. (2021) 56:593–619. doi: 10.1007/s00535-021-01788-x, PMID: 34231046 PMC8280040

[17] Tsochatzis EA Bosch J Burroughs AK. Liver cirrhosis. Lancet. (2014) 383:1749–61. doi: 10.1016/S0140-6736(14)60121-5 24480518

[18] Gines P Krag A Abraldes JG Solà E Fabrellas N Kamath PS. Liver cirrhosis. Lancet. (2021) 398:1359–76. doi: 10.1016/S0140-6736(21)01374-X 34543610

[19] Kondrup J Allison SP Elia M Vellas B Plauth M Educational and Clinical Practice Committee, European Society of Parenteral and Enteral Nutrition (ESPEN). ESPEN guidelines for nutrition screening 2002. Clin Nutr. (2003) 22:415–21. doi: 10.1016/s0261-5614(03)00098-0, PMID: 12880610

[20] Liang DQ Liu SJ. The relationship between stress level and physical exercise for college students. Chin Ment Health J. (1994) 01:5–6.

[21] Aubrey J Esfandiari N Baracos VE Buteau FA Frenette J Putman CT. Measurement of skeletal muscle radiation attenuation and basis of its biological variation. Acta Physiol (Oxf). (2014) 210:489–97. doi: 10.1111/apha.12224, PMID: 24393306 PMC4309522

[22] Hou L Deng Y Fan X Zhao T Cui B Lin L. A sex-stratified prognostic nomogram incorporating Body compositions for long-term mortality in cirrhosis. JPEN J Parenter Enteral Nutr. (2021) 45:403–13. doi: 10.1002/jpen.1841, PMID: 32359094

[23] Papadia C Osowska S Cynober L Forbes A. Citrulline in health and disease. Review on human studies. Clin Nutr. (2018) 37:1823–8. doi: 10.1016/j.clnu.2017.10.009, PMID: 29107336

[24] Rashid J Kumar SS Job KM Liu X Fike CD Sherwin CMT. Therapeutic potential of Citrulline as an arginine supplement: a clinical pharmacology review. Paediatr Drugs. (2020) 22:279–93. doi: 10.1007/s40272-020-00384-5, PMID: 32140997 PMC7274894

[25] Figueroa A Jaime SJ Morita M Gonzales JU Moinard C. L-Citrulline supports vascular and muscular benefits of exercise training in older adults. Exerc Sport Sci Rev. (2020) 48:133–9. doi: 10.1249/JES.0000000000000223, PMID: 32568925

[26] Newman JC Verdin E. Beta-Hydroxybutyrate: a signaling metabolite. Annu Rev Nutr. (2017) 37:51–76. doi: 10.1146/annurev-nutr-071816-064916, PMID: 28826372 PMC6640868

[27] Fukao T Mitchell G Sass JO Hori T Orii K Aoyama Y. Ketone body metabolism and its defects. J Inherit Metab Dis. (2014) 37:541–51. doi: 10.1007/s10545-014-9704-9 24706027

[28] Yao A Li Z Lyu J Yu L Wei S Xue L. On the nutritional and therapeutic effects of ketone body D-beta-hydroxybutyrate. Appl Microbiol Biotechnol. (2021) 105:6229–43. doi: 10.1007/s00253-021-11482-w, PMID: 34415393 PMC8377336

[29] Moller N Body K. 3-Hydroxybutyrate: minor metabolite - major medical manifestations. J Clin Endocrinol Metab. (2020) 105:2884–92. doi: 10.1210/clinem/dgaa370 32525972

[30] Poffe C Ramaekers M Van Thienen R Hespel P. Ketone ester supplementation blunts overreaching symptoms during endurance training overload. J Physiol. (2019) 597:3009–27. doi: 10.1113/JP277831, PMID: 31039280 PMC6851819

[31] Cox PJ Kirk T Ashmore T Willerton K Evans R Smith A. Nutritional ketosis alters fuel preference and thereby endurance performance in athletes. Cell Metab. (2016) 24:256–68. doi: 10.1016/j.cmet.2016.07.010, PMID: 27475046

[32] Dearlove DJ Harrison OK Hodson L Jefferson A Clarke K Cox PJ. The effect of blood ketone concentration and exercise intensity on exogenous ketone oxidation rates in athletes. Med Sci Sports Exerc. (2021) 53:505–16. doi: 10.1249/MSS.0000000000002502, PMID: 32868580 PMC7886359

[33] Juakiem W Torres DM Harrison SA. Nutrition in cirrhosis and chronic liver disease. Clin Liver Dis. (2014) 18:179–90. doi: 10.1016/j.cld.2013.09.004 24274873

[34] Heymsfield SB Arteaga C McManus C Smith J Moffitt S. Measurement of muscle mass in humans: validity of the 24-hour urinary creatinine method. Am J Clin Nutr. (1983) 37:478–94. doi: 10.1093/ajcn/37.3.478, PMID: 6829490

[35] Tosato M Marzetti E Cesari M Savera G Miller RR Bernabei R. Measurement of muscle mass in sarcopenia: from imaging to biochemical markers. Aging Clin Exp Res. (2017) 29:19–27. doi: 10.1007/s40520-016-0717-0 28176249

[36] Kashani KB Frazee EN Kukrálová L Sarvottam K Herasevich V Young PM. Evaluating muscle Mass by using markers of kidney function: development of the sarcopenia index. Crit Care Med. (2017) 45:e23–9. doi: 10.1097/CCM.0000000000002013 27611976

[37] Thongprayoon C Cheungpasitporn W Kittanamongkolchai W Harrison AM Kashani K. Prognostic importance of low admission serum creatinine concentration for mortality in hospitalized patients. Am J Med. (2017) 130:545–554.e1. doi: 10.1016/j.amjmed.2016.11.020, PMID: 27998681

[38] Dewulf JP Marie S Nassogne MC. Disorders of purine biosynthesis metabolism. Mol Genet Metab. (2022) 136:190–8. doi: 10.1016/j.ymgme.2021.12.016 34998670

[39] Emmanuel N Ragunathan S Shan Q Wang F Giannakou A Huser N. Purine nucleotide availability regulates mTORC1 activity through the Rheb GTPase. Cell Rep. (2017) 19:2665–80. doi: 10.1016/j.celrep.2017.05.043, PMID: 28658616

[40] Huang Z Zhang J Gu Y Cai Z Feng X Yang C. Research progress on inosine monophosphate deposition mechanism in chicken muscle. Crit Rev Food Sci Nutr. (2022) 62:1062–78. doi: 10.1080/10408398.2020.1833832, PMID: 33146022

[41] Bonagurio LP Murakami AE Moreira CA Comar JF Pozza PC. Dietary supplementation with inosine-5′-monophosphate improves the functional, energetic, and antioxidant status of liver and muscle growth in pigs. Sci Rep. (2022) 12:350. doi: 10.1038/s41598-021-04023-y, PMID: 35013384 PMC8748533

[42] Sahlin K Broberg S Ren JM. Formation of inosine monophosphate (IMP) in human skeletal muscle during incremental dynamic exercise. Acta Physiol Scand. (1989) 136:193–8. doi: 10.1111/j.1748-1716.1989.tb08652.x, PMID: 2782092

[43] Lounis MA Ouellet V Péant B Caron C Li Z al-Mass A. Elevated expression of Glycerol-3-phosphate phosphatase as a biomarker of poor prognosis and aggressive prostate cancer. Cancers (Basel). (2021) 13:1273. doi: 10.3390/cancers13061273 33805661 PMC8000625

[44] Possik E Madiraju SRM Prentki M. Glycerol-3-phosphate phosphatase/PGP: role in intermediary metabolism and target for cardiometabolic diseases. Biochimie. (2017) 143:18–28. doi: 10.1016/j.biochi.2017.08.001, PMID: 28826615

[45] Hernandez-Conde M Llop E Gómez-Pimpollo L Fernández Carrillo C Rodríguez L Van Den Brule E. Adding branched-chain amino acids to an enhanced standard-of-care treatment improves muscle Mass of cirrhotic patients with sarcopenia: a placebo-controlled trial. Am J Gastroenterol. (2021) 116:2241–9. doi: 10.14309/ajg.0000000000001301, PMID: 34074812

