# Supplementary material for: Differential metabolites in cirrhotic patients with hepatitis B and muscle mass loss
Source: Front Nutr. 2023 Feb 16;10:1068779. doi: 10.3389/fnut.2023.1068779 (PMC9980345; doi:10.3389/fnut.2023.1068779)
Supplement: Supplementary file 1 [file Data_Sheet_1.docx]

Supplemental Table 1. The metabolites in Group S and NS.

| **Metabolites** | **KEGG** | **VIP** | **FC** | ***P* value** |
| --- | --- | --- | --- | --- |
| L-kynurenine | C00328 | 3.639 | 0.063 | 0.023 |
| Inosine-5'-monophosphate | C00130 | 2.243 | 0.519 | <0.001 |
| Phosphoglycolic acid | C00988 | 2.101 | 0.541 | <0.001 |
| D-fructose-6-phosphate | C00085 | 1.648 | 0.675 | <0.001 |
| O-phosphoethanolamine | C00346 | 1.983 | 0.547 | <0.001 |
| Pyrophosphate | C00013 | 1.891 | 0.583 | <0.001 |
| N-acetylornithine | C00437 | 1.186 | 0.754 | <0.001 |
| Trehalose-6-phosphate | C00689 | 1.866 | 0.587 | <0.001 |
| Uridine 5'-monophosphate | C00105 | 1.634 | 0.656 | <0.001 |
| D-ribulose 5-phosphate | C00199 | 1.986 | 0.526 | <0.001 |
| Stigmasterol | C05442 | 1.471 | 0.606 | <0.001 |
| Lanosterol | C01724 | 1.800 | 0.581 | <0.001 |
| Fumaric acid | C00122 | 1.378 | 0.692 | 0.001 |
| Creatinine | C00791 | 1.468 | 1.664 | 0.001 |
| Guanosine | C00387 | 1.234 | 0.662 | 0.002 |
| N-acetyl-d-mannosamine | C00645 | 1.204 | 0.731 | 0.003 |
| 3-hydroxybenzoic acid | C00587 | 1.075 | 0.809 | 0.003 |
| Citrulline | C00327 | 1.585 | 0.639 | 0.003 |
| Glycolic acid | C03547 | 1.250 | 0.705 | 0.005 |
| Chenodeoxycholic acid | C02528 | 1.698 | 0.439 | 0.007 |
| (r)-3-hydroxybutyric acid | C01089 | 2.016 | 2.002 | 0.007 |
| Serotonin | C00780 | 2.274 | 0.448 | 0.009 |
| Docosahexaenoic acid | C06429 | 1.059 | 0.664 | 0.011 |
| L-tyrosine | C00082 | 1.160 | 0.661 | 0.011 |
| N-acetylglutamate | C00624 | 1.719 | 0.510 | 0.012 |
| Tartaric acid | C00898 | 1.174 | 0.620 | 0.013 |
| L-phenylalanine | C00079 | 1.211 | 0.740 | 0.016 |
| Homoserine | C00263 | 1.607 | 0.485 | 0.018 |
| N-acetyl-5-hydroxytryptamine | C00978 | 1.021 | 0.773 | 0.018 |
| Glycerol 3-phosphate | C00093 | 1.191 | 0.701 | 0.020 |
| 2-ketobutyric acid | C00109 | 1.732 | 2.189 | 0.020 |
| Quinic acid | C00296 | 1.741 | 0.621 | 0.021 |
| Spermine | C00750 | 1.350 | 0.510 | 0.023 |
| L-cystathionine | C02291 | 1.478 | 0.642 | 0.023 |
| L-glutamic acid | C00025 | 1.448 | 0.651 | 0.028 |
| Agmatine | C00179 | 1.021 | 0.789 | 0.039 |
| Dehydroascorbic acid | C05422 | 1.618 | 0.434 | 0.048 |

VIP: Variable importance on projection scores; FC: Fold change

Supplemental Table 2. Twenty-five pathways with *P* <0.05 and the related metabolites

| Pathway name | Pathway ID | Metabolites | *P* value |
| --- | --- | --- | --- |
| Arginine biosynthesis | hsa00220 | N-Acetyl-glutamate, Citrulline, L-glutamic acid, Fumaric acid, N-acetylornithine | <0.001 |
| Phenylalanine, tyrosine and tryptophan biosynthesis | has00400 | Quinic acid, L-phenylalanine, L-tyrosine, 3-hydroxybenzoic acid | <0.001 |
| Taste transduction | hsa04742 | Serotonin, Inosine-5'-monophosphate, L-glutamic acid | 0.0025 |
| Cocaine addiction | hsa05030 | L-glutamic acid, L-tyrosine | 0.032 |
| Central carbon metabolism in cancer | hsa05230 | D-fructose-6-phosphate, L-glutamic acid, Fumaric acid | 0.042 |
|  |  |  |  |
| Amphetamine addiction | hsa05031 | L-glutamic acid, L-tyrosine | 0.005 |
| Alcoholism | hsa05034 | L-glutamic acid, L-tyrosine | 0.005 |
| Gap junction | hsa04540 | L-glutamic acid, Serotonin | 0.006 |
| Arginine and proline metabolism | hsa00330 | Creatinine, L-glutamic acid, Spermine, Agmatine | 0.008 |
| Glutathione metabolism | hsa00480 | L-glutamic acid, Spermine, Dehydroascorbic acid | 0.008 |
| Oxidative phosphorylation | hsa00190 | Pyrophosphate | 0.013 |
| Butanoate metabolism | hsa00650 | (r)-3-hydroxybutyric acid | 0.015 |
| Glycine, serine and threonine metabolism | hsa00260 | 2-ketobutyric acid, Homoserine | 0.016 |
| Phenylalanine metabolism | hsa00360 | Fumaric acid, L-phenylalanine, L-tyrosine | 0.017 |
| Retrograde endocannabinoid signaling | hsa04723 | Trehalose-6-phosphate | 0.018 |
| Aminoacyl-tRNA biosynthesis | hsa00970 | L-glutamic acid, L-phenylalanine, L-tyrosine | 0.019 |
| Glyoxylate and dicarboxylate metabolism | hsa00630 | Phosphoglycolic acid | 0.031 |
| cAMP signaling pathway | hsa04024 | (r)-3-hydroxybutyric acid, Serotonin | 0.031 |
| Glucagon signaling pathway | hsa04922 | D-fructose-6-phosphate, L-glutamic acid, Fumaric acid | 0.031 |
| Renal cell carcinoma | hsa05211 | Fumaric acid | 0.033 |
| Parkinson disease | hsa05012 | Pyrophosphate | 0.033 |
| Cysteine and methionine metabolism | hsa00270 | 2-ketobutyric acid, Homoserine | 0.036 |
| Alanine, aspartate and glutamate metabolism | hsa00250 | L-glutamic acid | 0.038 |
| Pathways of neurodegeneration - multiple diseases | hsa05022 | Pyrophosphate, L-glutamic acid | 0.038 |
| Protein digestion and absorption | hsa04974 | L-glutamic acid, L-phenylalanine | 0.041 |
