# Supplementary material for: Differential metabolites in cirrhotic patients with hepatitis B and muscle mass loss
Source: Front Nutr. 2023 Feb 16;10:1068779. doi: 10.3389/fnut.2023.1068779 (PMC9980345; doi:10.3389/fnut.2023.1068779)
Supplement: Supplementary file 2 [file Data_Sheet_2.doc]

Supplement Figure 1. (A) Volcano plots of Groups S and H; (B) Volcano plots of Groups NS and H; (C) Correlation analysis of metabolites in Groups S and H; (D) Correlation analysis of metabolites in Groups NS and H; (E) Heatmap of the correlation analysis of the expression levels of metabolites in Groups S and H; (F) Heatmap of the correlation analysis of the expression levels of the metabolites in Groups NS and H
